# Degradation of 2,6-dicholorophenol by *Trichoderma longibraciatum* Isolated from an industrial Soil Sample in Dammam, Saudi Arabia

**DOI:** 10.1038/s41598-022-07016-7

**Published:** 2022-02-21

**Authors:** Amira H. Alabdalall, Fatimah A. Aldakheel, Ibtisam M. Ababutain, Hanen Chakroun, Azzah I. Alghamdi, Ines Hammami, Sahar K. Al Dosary, Tamer E. Youssef, Ahmed M. Albarrag, Sumayh A. Aldakeel, Rawan Aldughaish, Nada Al Qurin, Hesham M. ElKomy

**Affiliations:** 1 Department of Biology, College of Science, Imam Abdulrahman Bin Faisal University, P.O.Box 1982, Dammam, Saudi Arabia; 2 Basic and Applied Scientific Research Center (BASRC), Imam Abdulrahman Bin Faisal University, P.O. Box 1982, Dammam, 31441 Saudi Arabia; 3 Department of Pathology, School of Medicine, King Saud University, Riyadh, Saudi Arabia; 4 The National Center for Genomic Technology (NCGT), Life Science and Environment Research Institute, King Abdulaziz City for Science and Technology (KACST), Riyadh, Saudi Arabia; 5 Present Address: Polymer Synthesis Laboratory, KAUST Catalysis Center, Physical Sciences and Engineering Division, King Abdullah University of Science and Technology (KAUST), Thuwal, 23955 Saudi Arabia; 6 Present Address: Genomic of Infectious Diseases Laboratory, Saudi Center for Disease Prevention and Control, Public Health Authority, Riyadh, Saudi Arabia

**Keywords:** Biochemistry, Biological techniques, Biotechnology, Ecology, Microbiology

## Abstract

2,6-Dichlorophenol (2,6-DCP) is an aromatic compound with industrial importance in making insecticides, herbicides, and other organic compounds. However, it poses serious health and ecological problems. Microbial degradation of 2,6-DCP has been widely applied due to its effectiveness and eco-friendly characteristics. In this study, *Trichoderma longibraciatum* was isolated from an industrial soil sample in Dammam, Saudi Arabia using the enrichment method of mineral salt's medium (MSM) amended with 2,6-DCP. Morphological and molecular identification (using the internal transcribed spacer rRNA gene sequencing) of the 2,6-DCP tolerating fungal isolate were charactraized. The fungal isolate has demonstrated a tolerance to 2,6-DCP up to 300 mg/L. Mycelial growth and fungal sporulation were reduced with increasing 2,6-DCP concentrations up to 96 h incubation period. However, after 168 h incubation period, the fungal isolate recorded maximum growth at all the tested 2,6-DCP concentrations up to 150 mg/L. Carboxy methyl cellulase production by tested fungus was decreased by increasing 2,6-DCP concentration up to 75 mg/L. The biodegradation pattern of 2,6-DCP in GM liquid medium using GC–mass analysis as well as the degradation pathway was presented. This study provides a promising fungal isolate that could be used in the bioremediation process for chlorinated phenols in soil.

## Introduction

Chlorophenols (CPs) are aromatic compounds with at least one chlorine atom and one hydroxyl group^1^. These compounds are used as mothproofing agents, miticides, germicides, algicides, fungicides and wood preservatives, as well as in the synthesis of dyes and drugs^2, 3^. They are also discharged into the environment through industrial, urban and agricultural wastewaters. These halogenated organic products are source of pollution for soil and ground water with regard to their high toxicity, strong odor emission and persistence^4^. In addition, Cps are characterized by their carcinogenic, mutagenic, and cytotoxic effect on human as showed by the World Health Organization^5^.

The removal of chlorinated phenols from contaminated sites involves several chemical and physical methods including oxidation by chemical, photochemical or photocatalytic processes and adsorption respectively^4, 6^. But considering the hazardous byproducts, the high cost and the limited concentration range of these processes, attention has been focused on the environmentally safe, cost effective and efficient bioremediation methods^7^. To illustrate, bacterial species including genera of *Pseudomonas*, *Ralstonia*, *Rhodococcus*, *Alcaligenes* and *Herbaspirillum* are capable of utilizing CPs as sole carbon and energy sources^8^. Furthermore, white rot fungi are promising organisms because of their ability to completely degrade aromatic xenobiotics^7, 9^. This ability is associated with the production of extracellular oxidoreductases (laccases, peroxidases and tyrosinases) that can remove chlorophenols from wastewater through an oxidative coupling reaction. For instance, *Phanerochaete chrysosporium* has been shown to degrade chlorinated aromatic compounds such us 2–4 dichlorophenol, pentachlorophenol and 2,4,5-trichlorophenoxiacetic acid using a degradation system consisting of peroxidases, laccases, cellobiose dehydrogenase and transmembrane methyl transferase^10^. Members of the genus *Trametes* are also excellent candidates for these activities with confirmed bioremediation capacity^7, 11–13^. Fungi from other classes showed also various extent of chlorophenols degradation^13–15^. Although fungi of the genus *Trichoderma* have been studied mainly as biological control agent against plant diseases, species of *Trichoderma* has been shown to metabolize CPs. For instance, Cser-jesi and Johnson,^16^ reported the degradation of pentachlorophenol by *T. irgatum*. Furthermore, *T. harzianum* isolated from water samples of Lake Bonney, South of Australia mineralized a minor percentage (2–3%) of spiked radiolabeled pentachlorophenol and partially dehalogenated (46%) spiked tetrachloroguaiacol in mineral salts medium^17^. Additionally, PCP was completely degraded co-metabolically by *T. longibrachiatom* DSM 16517^18^. Chakroun et al*.*^19^, demonstrated the transformation of 2,4-dichlorophenoxyacetic acid and 4-chlorophenol in the presence of 2,2′-azinobis-(3-ethylbenzthiazoline-6- sulphonate) as mediator by the laccase purified from *T. atroviride*. The production of laccases by *Trichoderma* species is very interesting because it is known to produce a variety of cellulolytic enzymes including carboxymethyl cellulase (CMCase)^20, 21^. Therefore, the cooperation between lignocellulolytic enzymes could enlarge the potential applications of such strain in various industries and in the treatment of agricultural and industrial wastes.

The present study aimed to (i) isolate fungi from an industrial soil sample in Dammam, Saudi Arabia to screen these fungal strains for 2,6-DCP degradation, (ii) identify the isolated 2,6-DCP tolerant fungus molecularly, (iii) investigate the transformation pattern and pathway of 2,6-DCP and (iv) study the effect of 2,6-DCP on its growth and carboxy methyl cellulase activity .

## Materials and methods

### Isolation of 2,6-Dichlorophenol degrading fungi

Hydrocarbons polluted soil samples were collected from an automobile oil change station in industrial zones at Dammam city, Saudi Arabia, on 16 March 2019. One gram of polluted soil was inoculated into 100 ml of a Mineral Salt's Medium (MSM)^17^ containing (in grams of ingredient per liter): K_2_HPO_4_, 0.75; KH_2_PO_4_, 0.2; MgSO_4_, 0.1and FeSO_4_.7H_2_O, 0.06. The final pH was adjusted to 7.0^22^. The tested pollutant 2,6-Dichlorophenol(2,6-DCP) was added at the concentration 100 mg/L as sole carbon and energy source. The cultures were placed on a shaker (120 rpm) for a week at 30 ºC. Five ml of cultures fluid was subculture into fresh sterile MSM containing the tested 2,6-DCP. The pollutant 2,6-DCP concentrations were increased gradually: 0, 25, 50, 75, 100 and 150 mg/L. Fungal Colonies becoming visible after 3–4 days of incubation at 30 °C were picked up and sub-cultured to ensure purity. Pure cultures were grown in MSM containing 100 mg/L 2,6-DCP slants and stored at 4 °C for further investigations.

### Fungal characterization and identification

Fungal isolates that tolerated up 100 mg L^−1^ of 2, 6-DCP were characterized morphologically and molecularly. The morphological appearances of the selected fungal isolates grown on Potato Dextrose Agar (PDA) plates were characterized by visual observation and micro-morphological techniques^23^.

For molecular identification, genomic DNA was extracted from pure colonies of grown fungal mycelia using the column-based method of QlAamp DNA Mini Kit (QIAGEN, Germany) following manufacturer’s instructions with additional steps: (samples were initially treated with 0.5 mm glass beads and an incubation time at 56 °C for 45 min to ensure cells homogenization). The DNA quality and quantity were determined spectrophotometrically using NanoDrop, 2000c spectrophotometer (Thermo Scientific, USA).PCR reactions of 50 µl were performed including the following: (2X Go Taq Green Master Mix, Promega, USA) and 2 µM of the previously described primers; ITS1: (5′-CTT GGT CAT TTA GAG GAA GTA A-3′)^24^ and ITS4: (5′-TCC TCC GCT TAT TGA TAT GC-3′)^25^ to amplify the internal transcribed spacer (ITS) rRNA genes. PCR reaction was run under the following thermocycler parameters: an initial denaturation at 95 °C for 10 min, followed by forty-five cycles of: denaturation at 95 °C for 30 s, 55 °C annealing for 30 s, and 72 °C extension for 1 min, with a final extinction step at 72 °C for 10 min.

### PCR purification and sequencing

Amplicons obtained from previous PCR step were purified using ExoSAP-IT (Applied Biosystems, Thermo Fisher Scientific, USA) following manufacturer’s recommendations. Using 10 µM of either forward (ITS1) or reverse (ITS4) primer used in the initial amplification step, samples were amplified using BigDye Terminator v3.1 Cycle Sequencing Kit (Thermo Fisher Scientific, USA). Following the amplification,. Purification was conducted using Cycle sequencing purification- BigDye X-Terminator purification kit (Thermo Fisher Scientific, USA) to prepare the samples for sequencing, which were carried out using the series Genetic Analyzer 3500 (Thermo Fisher Scientific, USA).

### Radial fungal growth in solid medium with 2,6-DCP

The rate of fungal growth on PDA medium supplemented with various concentrations of 2,6-DCP (0, 25, 50, 75, 100 and 150 mg/L) was tested. Agar plates were inoculated with 5 mm plug of fungal mycelium and incubated at 28 °C. Four replicates were prepared for each concentration. Radial mycelial growth was measured after 1, 2, 3, 4 and 7 days of incubation^23^.

### Biodegradation of 2,6-DCP in GM liquid medium

Biodegradation of 2,6-DCP (0, 25, 50, 75, 100 and 150 mg/L) was determined in Glucose Minimal (GM) liquid medium described by^26^. The GM medium used contained (g/l): 1.0 K_2_HPO_4_, 0.01 ZnSO_4_.7H_2_O, 0.005 CuSO_4_.5H_2_O, 0.5 MgSO_4_.7H_2_O, 0.01 FeSO_4_.7H_2_O, 0.5 KCl, 10 glucose and 3.0 NaNO_3_ as an energy source of nitrogen. The pH of the liquid medium was adjusted to 5.5 before autoclaving at 121 °C for 15 min. Two pieces of agar plugs (5 mm in diameter) of a 4-day old fungal isolate grown on PDA at 28 °C were used as inoculums in each of the 20 mL liquid medium in 100 mL flasks. The cultures were grown under static condition for 12 day at 28 °C. A flask containing 2,6-DCP without fungal inoculum was prepared as control. All the experiments were done in triplicate. Fungal growth was estimated after filtration and overnight drying in an over at 70 °C. The experiments of biodegradation products were carried out with the culture filtrate of 2,6-DCP (100 mg/L) treatment after 12 days incubation period.

A Gas Chromatography-Mass Spectrometry (GC–MS) Model QP2010 SE (Shimadzu-Japan) was used to quantify the biodegradation of 2,6-DCP and its breakdown products. The GC–MS was outfitted with a 5 Sil MS capillary column with a length of 30 m, an internal diameter of 0.25 mm, and a film thickness of 0.25 m. Helium was used as the carrier gas at a flow rate of 2.5 ml min^–1^. The injector temperature and the ion-source temperature were set to 250 °C. The oven temperature was set to range from 50 to 300 °C, with a hold time of 3 min and a total run duration of 29 min. ACQ Mode Scan range: 35 m/z to 500 m/z, scan speed: 1666.

### Effect of 2,6-DCP on the production of Carboxymethyl Cellulase by fungal isolate

Carboxymethyl Cellulase (CMCase) production by fungal isolate and the effect of 2,6-DCP at different concentrations was assayed according to Dubois et al.^27^. Conidia of *Trichoderma* were produced by transferring agar discs (7 mm in diameter) from PDA culture to flasks each containing 20 ml of minimal synthetic medium (MSM) with the following composition (g/l): MgSO_4_.7H_2_O 0.2; K_2_HPO_4_ 0.9; KCl 0.2; NH_4_NO_3_ 1.0; FeSO_4_.7H_2_O 0.002; MnSO_4_ 0.002 and ZnSO_4_ 0.002. supplemented with 1% CMCase and incubated at 30 °C for 7 days. Enzyme activity was estimated by measuring the accumulation of soluble sugars using the phenol sulphuric acid as following: 0.5 ml of the diluted glucose standard was added to each tube along with 0.5 ml at 5% phenol solution. Likewise, 0.5 ml of the sample's supernatant was added to each respective tube with 0.5 ml of 5% phenol solution. All tubes were vortexed to thoroughly mix the contain 2.5 ml of Conc. H_2_SO_4_ was added to each tube. The tubes were sealed and were incubated for 30 min at room temperature before reading the absorbance at 485 nm. After 7-day incubation period, the fungal mycelia were filtrated dried at 80 °C for constant weight. The fungal growth was reported as mg/20 ml.

### Phylogenetic analysis

Nucleotide sequences of the internal transcribed spacer (ITS) rRNA gene region of the fungi known for their 2,6-DCP degredation were retrieved from the DNA database of GenBank (www.ncbi.nlm.nih.gov/genbank/). The determined nucleotide sequences of the isolated fungi in this study and other fungi with 2,6-DCP degredation caractaristics were subjected to multiple nucleotide sequence alignment using Cluster Omega tool (http://www.ebi.ac.uk/Tools/msa/clustalo/). From the multiple alignment data, a phylogenetic tree was constructed using Interactive Tree Of Life (iTOL) tool (Letunic and Bork^28^ Nucleic Acids Res https://doi.org/10.1093/nar/gkab301) to analyze the relationship between the fungi species. Tree construction was performed using the neighbor joining method and distance divergence (%) was determined using the Molecular Evolutionary Genetics Analysis 7 (mega7) software package^29^.

### Statistical analysis

Data presented in this study were means of three replications, One-way ANOVA test was used in Statistical Package for Software Science (SPSS) statistic program at *P* < 0.001. Statistical data was analyzed using Statistical Packages for Software Science^30^ version 21 Armonk, New York, IBM Corporation. The R programming language (RStudio) (https://www.rstudio.com/) was used to develop analytics and visualizations graphics.

### Ethical approval

This article does not contain any studies with human participants or animals performed by any of the authors.

## Results

### Fungal isolation and identification

**Figure 1:**
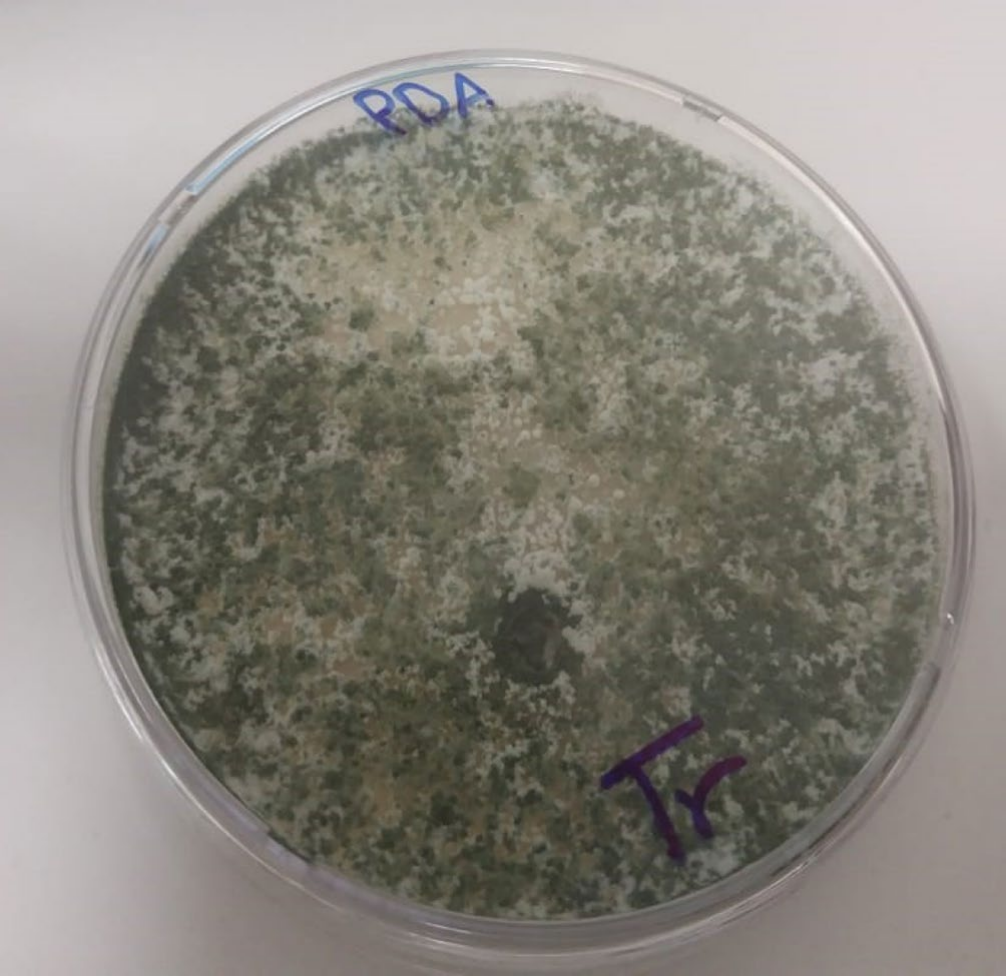
Growth of *T. longibraciatum* on PDA medium after 4-day incubation period.

Isolation results indicated that only one fungal strain could survive in the presence of 300 mg/L of 2,6-DCP in the media. Cultural and morphological characterization of the 2,6-DCP tolerant fungal isolate showed that it was belonging to the genus *Trichoderma* (Ascomycetes). Fast growing colony that fills up 9 cm Petri dish in 4–5 days. Cottony mycelial mat formed were white at first and becoming dark grey with conidiospores development (Fig. 1).

**Figure 2:**
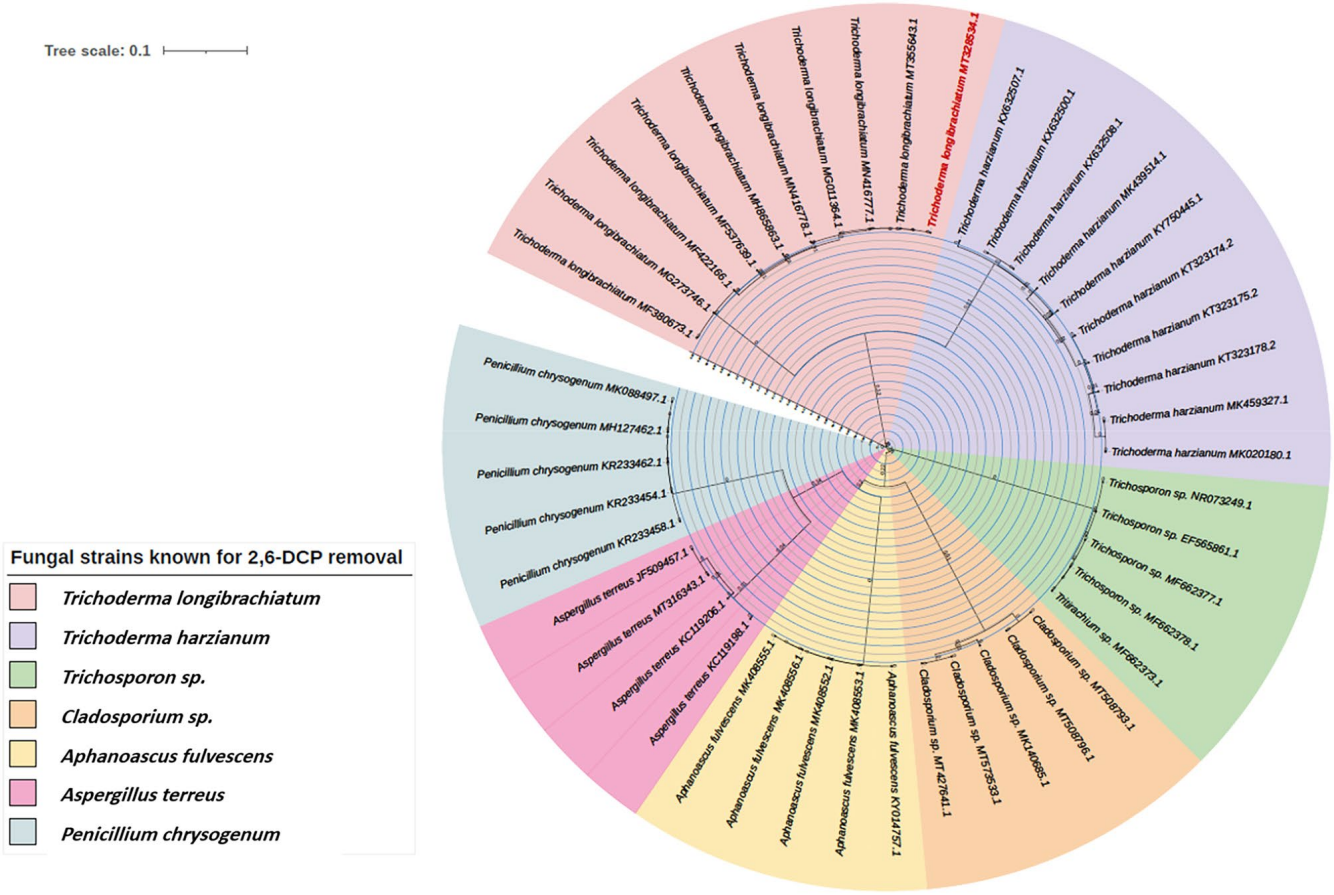
Neighbour-joining tree from ITS sequences showing the relationship between the known 2,6-DCP tolerant fungus, sequences of the isolates retrieved from the GenBank database.

Sequence-based molecular identification of the fungal isolate further confirmed as *Trichoderma longibraciatum*.The consensus sequence of ITS gene was submitted to NCBI GenBank record with accession number of MT328534. The phylogenetic tree constructed based on the ITS sequences of the new *Trichoderma* isolate and other fungal strains known for their 2,6-DCP tolerant and removel caractaristics. Phylogenetic analysis revealed the genetic relationship of the new isolate to the other fungal strains where it is closely related to *Trichoderma harzianum* (95% of sequence identities) followed by *Cladosporium sp.* (62% of sequence identities) and highly diverse from *Trichosporon sp.* (36% of sequence identities) (Fig. 2).

### Radial growth of T. longibraciatum in solid medium with different concentrations of 2,6-DCP

**Figure 3:**
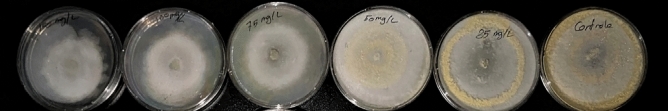
Growth and sporulation of *T*. *longibraciatum* isolate on PDA containing 2,6-DCP at different concentrations (150, 100, 75, 50, 25, and 0 mg/L), after 4 days of incubation period.

**Figure 4:**
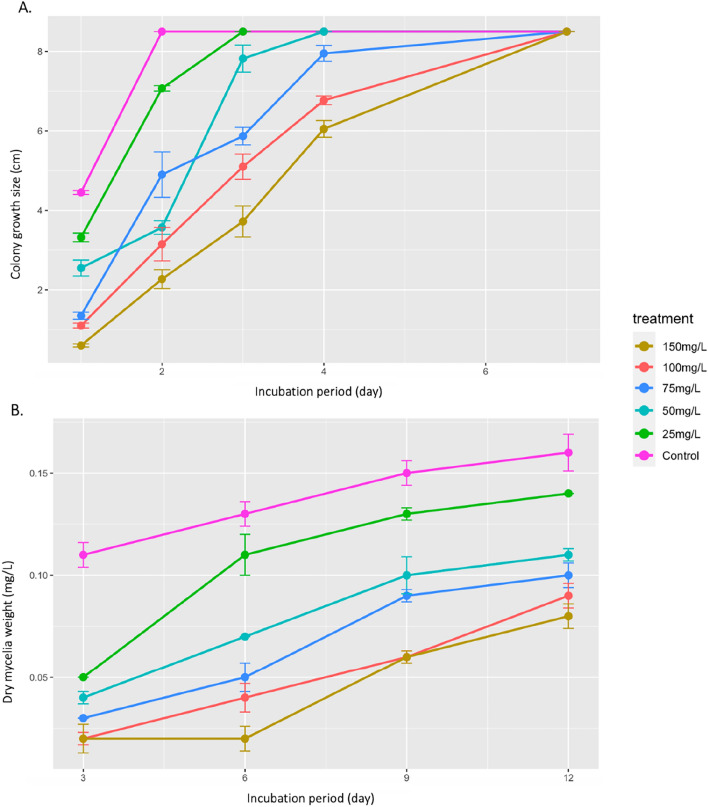
(**A**) *T. longibraciatum* growth on PDA containing 2,6-DCP at different concentrations, colony growth size (cm) mean ± SD. (**B**) Dry mycelia weight (mg/L) of *T. longibraciatum* grown in GM medium at different 2,6-DCP concentrations.

A fungal isolate *T.longibraciatum* was screened for its ability to grow and tolerate 2,6-DCP at different concentration ranging from 25 to 150 mg/L on solid agar plate (Fig. 3). Results showed that fungal isolate exhibited different degrees of sensitivity towards 2,6-DCP up to 4 days incubation period. The mycelial growth as well as fungal sporulation were reduced with increasing the 2,6-DCP concentrations compared with control. However, increasing the incubation period up to 7 days significantly reduced the toxicity effect of the 2,6-DCP in all concentrations (Fig. 4A).

### Biodegradation of 2,6-DCP in GM liquid medium

Estimate the dry fungal growth in presence of 2,6-DCP.

Based on the present results, *T. longibraciatum* was able to tolerate 2,6-DCP up to 150 mg/L after incubation for 12 days (Fig. 4B). Fungal dry weight was decreased by increasing 2,6-DCP concentration to reach the highest weight at 0.14 mg/L of (25 mg/L) 2,6-DCP concentration, and the lowest weight at 0.08 mg/L of (150 mg/L) 2,6-DCP tested concentration. Fungal dry weight in the control showed higher fungal dry mass combared to the tested 2,6-DCP concentrations and reached the highest weight after 12 days of incubation (0.16 mg/L).

### Gas chromatography/mass spectrometry (GC/MS) analysis

**Figure 5:**
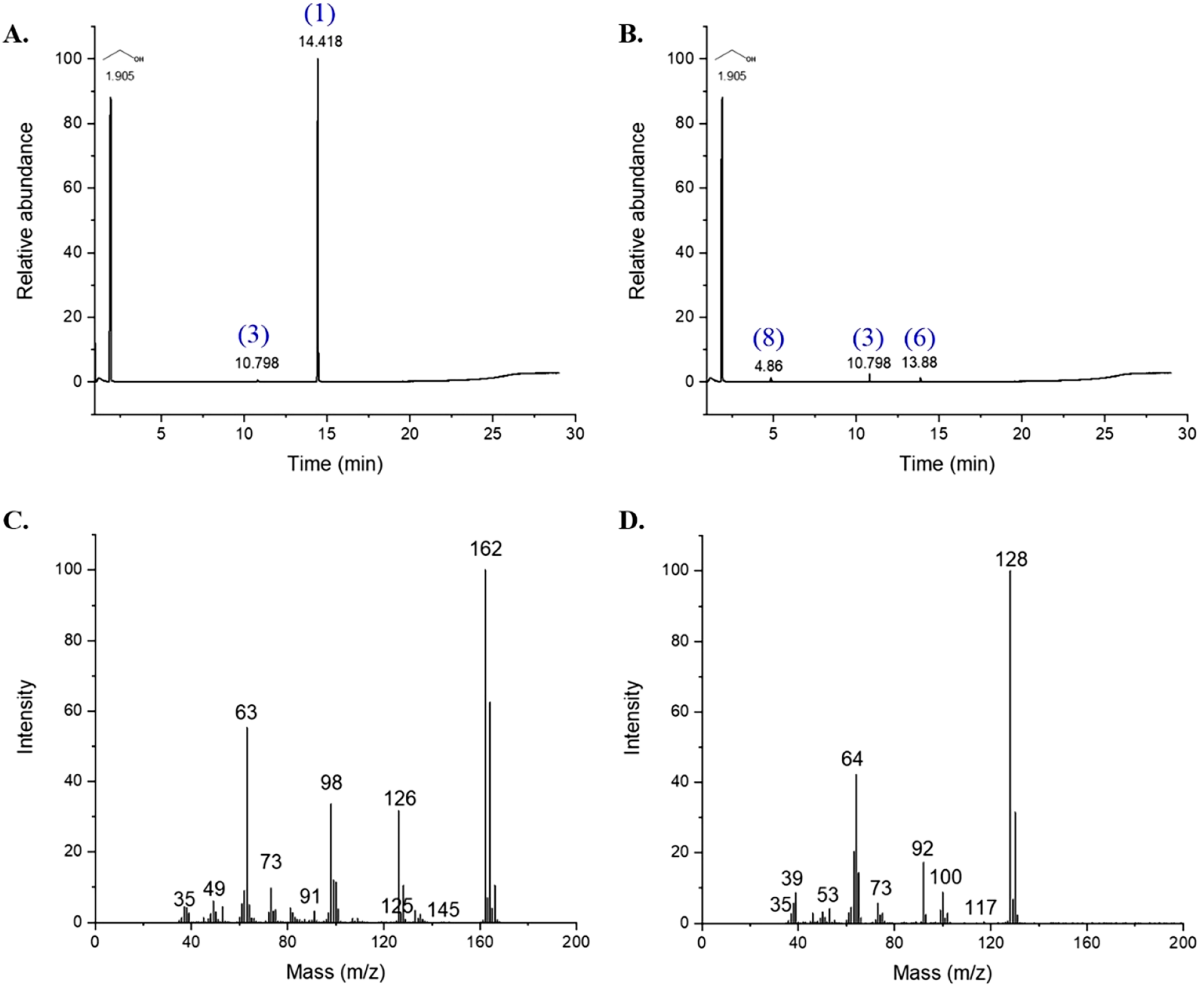
GC–MS analysis (**A**) Gas chromatography of 2,6-DCP the authentic sample (control), (**B**) Gas chromatography of the extracted bio-treated sample obtained during 2,6-DCP biodegradation by *Trichoderma longibraciatum* treated medium amended on the 12th day, (**C**) and (**D**) corresponding mass spectrum of the peak appearing at the chromatogram (**A**) and (**B**), respectively.

**Figure 6:**
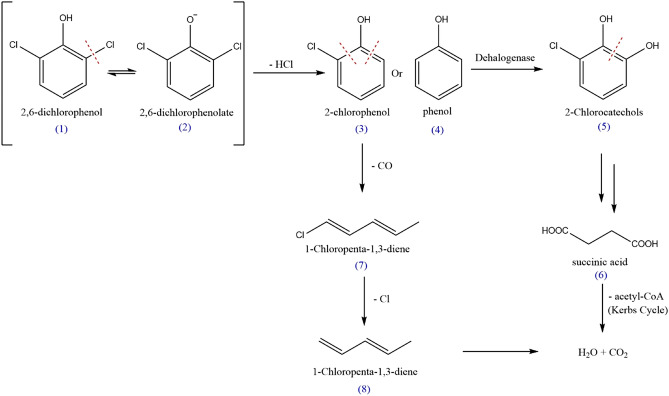
Proposed pathway of the degradation of 2,6-DCP by *Trichoderma longibraciatum.*

The release of one chloride ions per mol of 2,6-DCP during the biodegradation was indicated in Fig. 5B and supported by the proposed mechanism of 2,6-DCP degradation (Fig. 6). GC–MS shown the peak correspond to 2,6-DCP, with the molecular ion peak at m/z 163 [M]^+^ (1) at retention time 14.4 min, and its fragments at m/z 162 [M-1]^+^ (2), 128 [M-Cl]^+^ (3), and 92 [M-2Cl]^+^(4) at retention time 12.01 min. Other fragments were found at m/z 145, 117, 100, and 65 with their relative intensities as [CP-Cl + OH] (5), [HOOCCH_2_CH_2_COOH] (6) [CP-CO]^+^(7), and [CP-CO-Cl]^+^(8), respectively (Figs. 5, 6).

### Effect of 2,6-DCP on carboxymethyl cellulase production by fungal isolate

Data in Table 1 show the results of the effect of different 2,6-DCP concentrations on carboxymethyl cellulase production by fungal isolate and dry weight. Results showed that enzyme production was decreased by increasing 2,6-DCP concentration. Maximum inhibitory action was achieved at the concentration of 100 mg/L2,6-DCP. However, fungal isolate dry weight was not affected by 2,6-DCP at all concentrations.

**Table 1:** Carboxymethyl cellulase (CMC-ase) production by fungal isolate grown under different 2,6-DCP concentrations, after 7 days of incubation.

2,6-DCP (mg/L)	CMC-ase (U/mL)	Inhibition %	Fungal dry weight (g/20 mL)
Control	1.54 ± 0.14	–	0.37 ± 0.01
25	1.41 ± 0.05	12.7	0.39 ± 0.01
50	1.42 ± 0.06	13.9	0.51 ± 0.01
75	0.22 ± 0.01	86.6	0.37 ± 0.01
100	0.0 ± 0.00	100	0.53 ± 0.01
150	0.0 ± 0.00	100	0.48 ± 0.01

Results are represented as means ± standard error (SD) of three replicates.

## Discussion

The removal of dichlorophenol compounds, which are toxic, hardly degraded, and tend to accumulate in soil and water, has been primerily achieved using physical and chemical methods^31^. The disadvantages of these methods, such as the potential of forming hazardous byproducts, have been extensively demonstrated^32, 33^. On the other hand, biological degradation methods are considered an attractive alternative phenol elimination approach. Bioremediation and phytoremediation exploiting microbes are innovative technologies that can alleviate a variety of environmental pollution problems. Fungi have been found to be effective in the degradation of chlorophenol found in polluted sites. In particular, *Trichoderma sp.* is one of the most effective groups of fungi employed in bioremediation^34–36^.

In light of the importance of exploring biological treatment alternatives agents, the current study aimed to investigate the ability of the isolated fungi in degrading 2,6-DCP and study the transformation and pathway patterns of 2,6-DCP. Moreover, the effect of 2,6-DCP on the fungal growth and carboxy methyl cellulase activity was investigated. Using morphological and molecular identifications, we determined that the isolated tolerant fungus strain reported in our investigation belonged to *Ttrichoderma longibraciatum*.

*T. longibraciatum* ability to grow and tolerate 2,6-DCP at various concentrations ranging from 25 to 150 mg/L was also examined. Fungal growth was determined by the radial growth and the dry weight of *T. longibraciatum* to predict the impact of 2,6-DCP concentrations. Findings demonstrated that the isolated fungi exhibited different degrees of sensitivity towards 2,6-DCP during the first 4 days incubation period, with a reduction in the growth associated with elevated 2,6-DCP concentrations compared with control. The inhibition of the growth may be attributed to the reduction of the ergosterol, a derivative of cholesterol essential for the regulation of fungal membrane fluidity and integrity targeted by fungicide^37^. Although the growth was reduced, the *T. longibraciatum* exhibited controlled growth after 7 days, even with the highest tested concentrations. Our results are supported by previous studies, which have demonstrated the ability of microorganisms to utilize the degraded chlorophenols as their sole carbon and energy source^38, 39^.

Chlorophenols compounds are degraded and transformed in both abiotic and biotic environments^39^. Despite the fact that chlorophenol compounds are poorly biodegradable and persistent in the environment, various studies have shown that aerobic breakdown of chlorophenol congeners is possible^38^. Oxidase must be engaged in the aerobic breakdown of chlorophenols for bacteria to integrate ambient oxygen into their substrates^40–43^. *T. longibraciatum longibraciatum* was incubated to 2,6-DCP for 12 days in order to confirm the degradation intermediates and hypothesize the degradation routes. The mass spectrum obtained for the control sample of 2,6-DCP in the positive-ion mode is illustrated in Fig. 5C. It gives molecular ion peak at m/z 162 with three other fragmented peaks in mass spectra at m/z 126, 98, and 63. Other fragments were found at m/z 145, 125, 91, 73, 49, 35 etc. The GC–MS spectra of the bio-treated sample differed from the fragmentation pattern of the control sample due to the loss of one Cl^−^ ion (Fig. 5D). It demonstrated the loss of a molecular ion peak at m/z 162 and the formation of a new molecular ion peak at m/z 128 along with three additional fragmented peaks in mass spectra at 100, 92, and 64 m/z.

Based on the intermediates identified in GC–MS, the underlying degradation pathways were listed in Fig. 6. The hypothesized route of 2,6-DCP breakdown by *Trichoderma longibraciatum* which reflects the majority of the compounds observed by GC–MS. The initial pathway for 2,6-DCP degradation, OH° radicals attack the aromatic ring and simultaneously release chlorine ions into solution and [M-Cl]^+^, this compound was clearly observed in Fig. 5B, as well as releasing [M-2Cl]^+^, and [CP-Cl + OH] products. In the second and third routes, 2,6-DCP is continually attacked by hydroxyl radicals, resulting in the formation of certain intermediates such as [CP-CO]^+^, [CP], and [CP-CO–Cl]^+^, the latest was observed in Fig. 5B. The hydroxylation of the aromatic ring breaks the ring, and the major results are acids such as succinic acid Fig. 5B. These intermediates eventually degrade into products absorbed into the cell's core metabolism such as carbon dioxide and water^44–46^.

Here we investigated the use of tolerant fungus strain isolated from industrial soil sample in degrading 2,6-DCP. Findings revealed that *T. longibraciatum* has the ability to tolerate high concentrations of 2,6-DCP; while also using it as an energy source. In addition, a potential pathway of 2,6-DCP degradation was proposed. The resistance of *T. longibraciatum* to high concentrations of 2,6-DCP, and its ability to degrade this hazardous and toxic compound makes it an essential fungal genus to be explored and employed in bioremediation of pesticide-contaminated sites. It is critical to investigate the production of oxygenases by strains known for their ability to tolerate evaluated levels of 2,6-DCP.

## Data Availability

The following information was supplied regarding data availability: The raw measurements are available in the Supplemental Files.
